# Myocardial inflammation and energetics by cardiac MRI: a review of emerging techniques

**DOI:** 10.1186/s12880-021-00695-0

**Published:** 2021-11-08

**Authors:** Vasiliki Tsampasian, Andrew J. Swift, Hosamadin Assadi, Amrit Chowdhary, Peter Swoboda, Eva Sammut, Amardeep Dastidar, Jordi Broncano Cabrero, Javier Royuela Del Val, Sunil Nair, Robin Nijveldt, Alisdair Ryding, Chris Sawh, Chiara Bucciarelli-Ducci, Eylem Levelt, Vassilios Vassiliou, Pankaj Garg

**Affiliations:** 1 Norwich Medical School, University of East Anglia, Norwich, UK; 2 Department of Infection, Immunity and Cardiovascular Disease, University of Sheffield, Sheffield, UK; 3 Leeds Institute of Cardiovascular and Metabolic Medicine, University of Leeds, Leeds, UK; 4 University of Bristol, Bristol, UK; 5 Cardiothoracic Imaging Unit, Hospital San Juan de Dios, Ressalta, HT Medica, Cordoba, Spain; 6 Norfolk and Norwich University Hospital, Norwich, UK; 7 Cardiology Department, Radboudumc, Nijmegen, The Netherlands

**Keywords:** Cardiovascular magnetic resonance (CMR), Ultrasmall superparamagnetic particles of iron oxide (USPIO), Magnetic resonance spectroscopy (MRS)

## Abstract

The role of inflammation in cardiovascular pathophysiology has gained a lot of research interest in recent years. Cardiovascular Magnetic Resonance has been a powerful tool in the non-invasive assessment of inflammation in several conditions. More recently, Ultrasmall superparamagnetic particles of iron oxide have been successfully used to evaluate macrophage activity and subsequently inflammation on a cellular level. Current evidence from research studies provides encouraging data and confirms that this evolving method can potentially have a huge impact on clinical practice as it can be used in the diagnosis and management of very common conditions such as coronary artery disease, ischaemic and non-ischaemic cardiomyopathy, myocarditis and atherosclerosis. Another important emerging concept is that of myocardial energetics. With the use of phosphorus magnetic resonance spectroscopy, myocardial energetic compromise has been proved to be an important feature in the pathophysiological process of several conditions including diabetic cardiomyopathy, inherited cardiomyopathies, valvular heart disease and cardiac transplant rejection. This unique tool is therefore being utilized to assess metabolic alterations in a wide range of cardiovascular diseases. This review systematically examines these state-of-the-art methods in detail and provides an insight into the mechanisms of action and the clinical implications of their use.

## Background

With more research focus being shifted in the complex pathophysiology of cardiovascular disease it is of paramount importance that we have the appropriate diagnostic techniques to identify the complicated disease processes that lie behind the symptoms. Cardiac energy metabolism in cardiovascular disease represents the foundation of the pathophysiological mechanisms that lie behind the disease processes and phenotypes and can guide the treatment strategy. Better understanding of cardiac metabolism and energetics can aid the diagnosis, prevention and management of each specific disease process. Cardiovascular Magnetic Resonance (CMR) has proved to be a powerful tool for this exact purpose with novel methods allowing for early accurate diagnosis and establishing appropriate therapeutic targets in a timely manner. In this review, we illustrate the critical role of state-of-the-art CMR methods in the assessment of inflammation and myocardial energetics in a variety of cardiovascular diseases.

### Ultrasmall superparamagnetic particles of iron oxide CMR for myocardial inflammation

Inflammation is an expanding risk factor for cardiovascular disease [1] and involved in a plethora of conditions including atherosclerosis [2], myocardial infarction [3] cardiomyopathies [4, 5], myocarditis [6] and valve disease [7]. With increasing advancements, CMR has enabled non-invasive assessment of inflammation at a myocardial level using short τ inversion recovery (STIR) sequences, late gadolinium enhancement (LGE), T1 and T2 mapping, negating the need for invasive myocardial biopsy in many patients. However, such sequences do not allow us to detect cellular inflammation. At this cellular level, macrophages play a major part in both the initiation and maintenance, as well as resolution of inflammation [8]. Identifying therefore macrophage activity is crucial as not only can it allow accurate diagnosis and monitoring of disease progression, but importantly it can enable targeted therapeutic interventions, both on macrophages and their by-products. This can allow control of the inflammation and thus slowing down, or even halting completely, specific cardiovascular disease. More recently, Ultrasmall superparamagnetic particles of iron oxide (USPIO) have been used successfully to assess cellular inflammation safely [9]. USPIO consist of nanoparticles with a diameter of < 50 nm which have the ability to be taken up by tissue-resident macrophages and neutrophils [3, 10]. These particles shorten the T2* relaxation time of tissues in which they acscumulate and therefore their impact can be assessed in T2*-weighted imaging and quantified by measuring the changes in the T2* and R2* values (R2* = 1/T2*) [10, 11]. Recently, the USPIO methodology has been validated with histological confirmation that USPIO accumulates in active cardiac macrophages and identifies areas of active cardiac inflammation [11]. The pathophysiology of USPIO and how USPIO CMR can be undertaken has been extensively reviewed elsewhere [3] and in this section we will focus on the potential clinical applications.

**Fig. 1:**
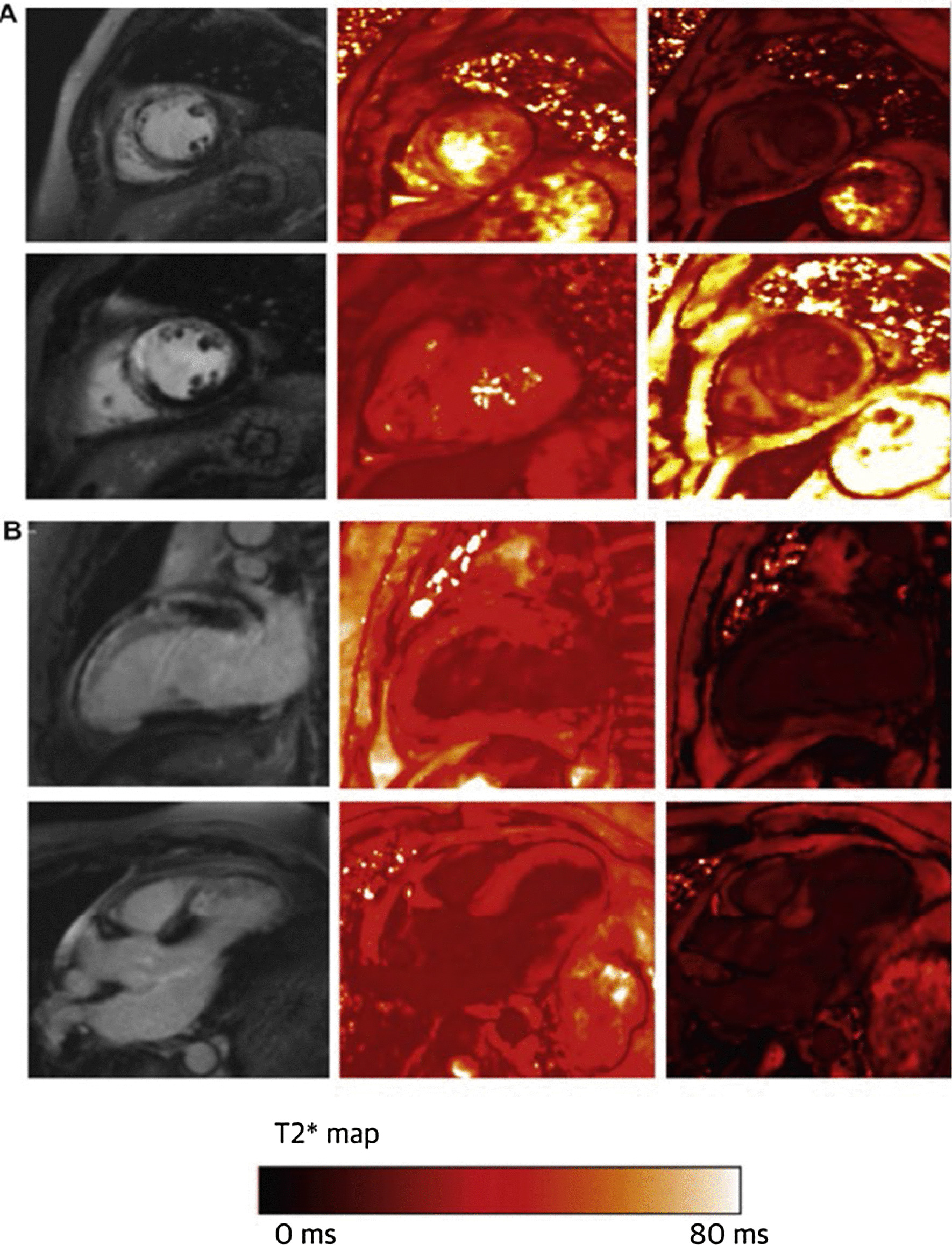
**a** Patient 1 week post left anterior descending artery infarction with extensive, anterior wall, transmural, mid-ventricle late gadolinium enhancement (LGE) on T1-weighted images (left column), homogeneous myocardial T2 ∗ values before erumoxytol (middle column), but intense dark ferumoxytol uptake in the region of the infarction 24 h post ferumoxytol infusion (right column). **b** Same patient with anteroapical, transmural LGE, again homogeneous T2 ∗ myocardial values at base, but clear ferumoxytol uptake on T2 ∗ scanning in the region of the LGE 24 h after ferumoxytol infusion. Image reproduced with permission from Merinopoulos et al. [10]

Multiple studies have assessed the role of USPIO CMR in STEMI or NSTEMI [11–15] and even following coronary artery bypass graft (CABG) [16]. These studies showed an increase in USPIO R2* in the infarcted and peri-infarct myocardium and this could be seen up to two weeks from the acute event (see Fig. 1). Some also showed that even the remote myocardium had a smaller increase in USPIO R2* lending therefore support that macrophage activation in the remote myocardium can effectively lead to myocardial damage. Interestingly, the study on CABG patients did not reveal significant differences in USPIO R2* indicating that perhaps the myocardial injury following CABG is less likely to be mainly driven by inflammation [16].

Whilst the role of oedema in Takotsubo cardiomyopathy had been recognized [17], one clinical study utilizing USPIO CMR enabled us to confirm for the first time that macrophage activity is the driving force. Fifty-five patients with acute Takotsubo cardiomyopathy were included as well as controls, and their USPIO CMR acutely showed greater differences between pre and post USPIO T2* in both the ballooning as well as the non-ballooning myocardial segments. At five months, this difference was no longer seen, indicating complete recovery of cellular inflammation. Interestingly, the patients also underwent ^31^P-CMR spectroscopy, which showed markedly reduced energetic state acutely, which however did not return to normal even at five months. The combination of the two methods allowed us to identify the cause of the acute decompensation (cellular inflammation) but also appreciate why some patients with Takotsubo cardiomyopathy continue to have long-lasting symptomatic and functional impairment (decreased energetics) [18] thus enabling to focus further research either in managing inflammation during the acute phase, or the decreased energetics in the longer term.

**Fig. 2:**
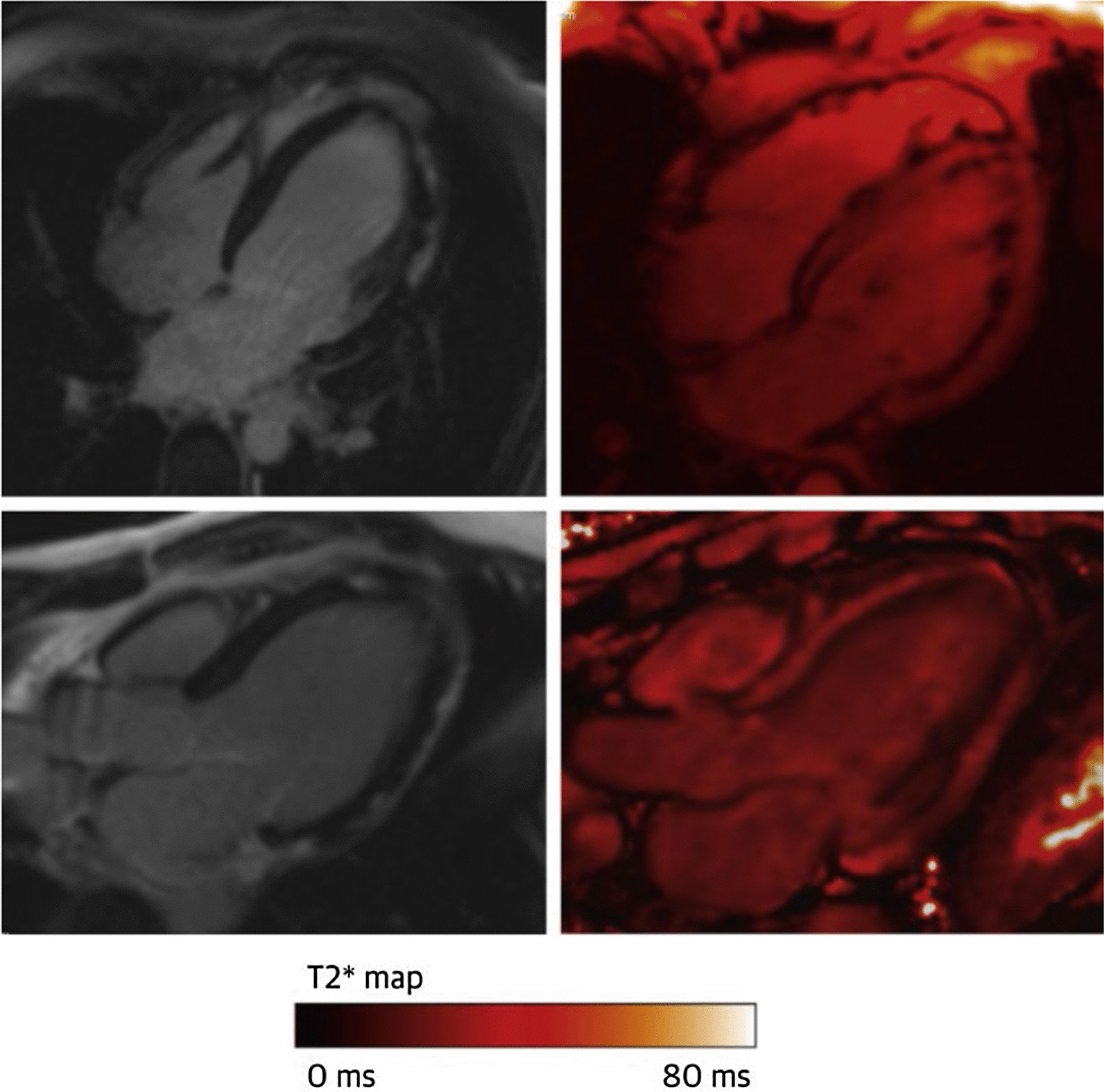
An example of a patient with acute myocarditis showing sub-epicardial LGE, inferiorly and inferolaterally on 4-chamber and 3-chamber views (left) but no evidence of ferumoxytol uptake within the regions displaying LGE 24 h following infusion (right). CMR = cardiac magnetic resonance; LGE = late gadolinium enhancement. Image reproduced with permission from Merinopoulos et al. [10]

The role of inflammation in myocarditis is well acknowledged, and indeed reflected in the recent Expert Recommendation with the Revised Lake Louise Criteria [19] whereby the oedema sequences, LGE and multiparametric parametric mapping play a crucial role in the diagnosis. However, cellular inflammation in myocarditis has been less well established or studied. The largest USPIO CMR study of acute myocarditis included only nine patients [20] with imaging results as shown in Fig. 2, but it failed to reveal any differences in USPIO R2* between the patients and controls, even when only the LGE positive myocardial areas were considered. Similarly, a much smaller study, including only five patients with acute myocarditis [21] likewise failed to show any significant differences compared to controls in USPIO R2*/R1. This is perhaps unexpected and would indicate that macrophages are not the primary medium of inflammation in myocarditis, unlike reported previously by histological studies which confirmed predominantly macrophage-rich inflammation associated with myocyte damage in areas of LGE [22]. It should be considered however, that even between them, the two studies only included 14 patients. Coupled with the known blooming artefacts which are more prominent in the inferolateral myocardium, which is the most common site of myocarditis, it might still be a possibility that USPIO simply failed to detect macrophage activity and further work is warranted.

The potential role of inflammation in triggering an ischaemic cardiomyopathy has long been considered [23]. However, many studies targeting inflammation therapeutically in this setting have been negative. Could inflammation however still play a role in non-ischaemic cardiomyopathy? A recent study involving only seven patients, studied with USPIO CMR on average 1.4 years following their acute myocardial infarction showed that both infarcted and non-infarcted myocardium had higher R2*. This would lend support to the notion that inflammation could possibly explain why only some patients progress to severe cardiomyopathy and others do not, even with similar index coronary events.

**Fig. 3:**
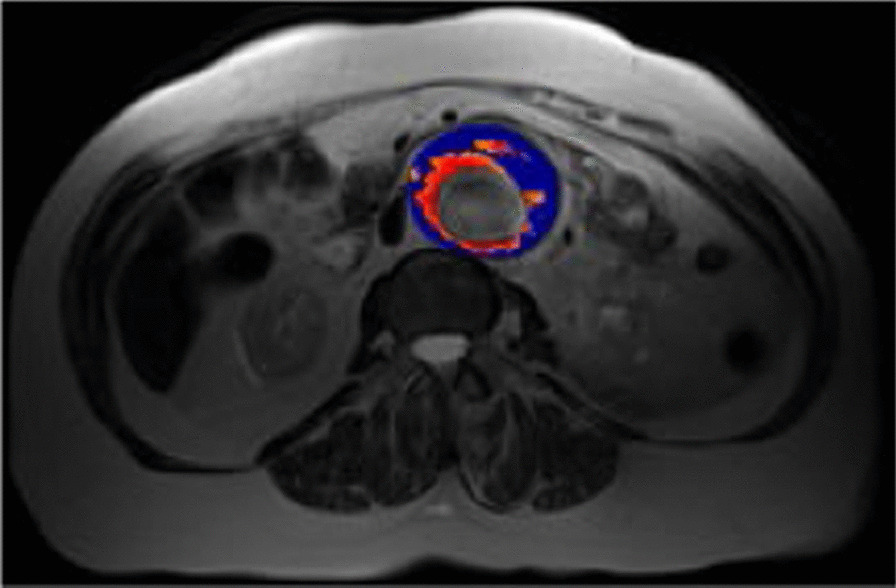
Classification of aneurysms based on uptake of ultrasmall superparamagnetic particles of iron oxide (USPIO). MRI is performed at baseline then 24 h following intravenous administration of USPIO. USPIO causes a reduction in T2* and can be quantified by comparing co-registered T2* images pre- and post- USPIO administration, presented as change in T2* represented as colour maps (as above). 'Positive uptake' of USPIO is denoted by the red colour (thresholded at change in T2* of at least 71% between pre-and post-USPIO administration), whereas blue denotes areas of no positive uptake. Of note, ‘significant’ uptake (i.e. USPIO positive) is defined as at least one focal area of USPIO uptake corresponding to 10 or more contiguous voxels of positive signal change at the aneurysm wall; USPIO uptake at the periluminal area is not thought to be clinically significant. Image courtesy of Dr Rachel Forsythe, University of Edinburgh

Multiple studies utilising USPIO MRI have investigated the role of inflammation in carotid atherosclerosis, aortic aneurysms and even monitor the effect of statins on atherosclerosis regression [24, 25]. These studies confirmed macrophage activity in carotid arteries which appeared independent from the degree of stenosis, suggesting therefore that both are independent risk factors. In addition, high dose statin therapy also showed a reduction in USPIO-defined inflammation in the carotids, indicating that USPIO can also potentially act as the investigation of choice for early endpoints. Whether USPIO and ^18^F-FDG correlate well, or whether they might indicate different parts of inflammation remains unclear as to date the studies are conflicting [26, 27]. USPIO MRI can be helpful in other vascular structures such as the abdominal aorta (see Fig. 3), where it can potentially be used to identify patients at higher risk of aneurysmal expansion (than based on diameter alone) and in turn inform the need and timing for surgery [28].

The clinical application of USPIO is further promoted by their safety profile. It has been demonstrated that it is a safe alternative to gadolinium-based contrast agents in patients with chronic kidney disease at risk for nephrogenic systemic fibrosis, and therefore can safely be used in this patient cohort [29]. Perhaps one of the main safety concerns is the risk of acute hypersensitivity allergic reaction, a rare but potentially life-threatening complication for which the U.S. Food and Drug Administration issued a boxed warning about the use of ferumoxytol a few years ago [30]. Since then, however, the safety profile of these agents has further improved, and large studies have successfully demonstrated and support their safe use in clinical practice [9, 31].

In summary therefore, USPIO MRI is an emerging safe method, to assess cellular inflammation. Whilst at the moment its role is mainly limited to research, future applications could include diagnostic as well as serial imaging applications, monitoring the response to treatment, or even guiding the timing of intervention.

### Phosphorus magnetic resonance spectroscopy and myocardial energetic impairment

Magnetic resonance spectroscopy (MRS) is an optimal non-invasive tool used commonly to analyse cardiac energy metabolism both in the clinical and pre-clinical setting. In the heart, ATP delivery can occur through the creatine kinase (CK) system, which catalyzes the following reversible reaction: Phosphocreatine + ADP + H +  ↔ Creatine + ATP. The relative concentration of phosphocreatine to ATP (PCr/ATP) is a marker of the myocardium’s ability to convert substrate into ATP for active processes, and a representantive index of the myocardial energetic state. Phosphorus magnetic resonance spectroscopy (^31^P-MRS) allows accurate—yet non-invasive—evaluation of the myocardial PCr/ATP ratio [32] as well as absolute levels of high-energy phosphates [33]. Using this technique, studies have shown myocardial energetic compromise to be a feature of diabetic cardiomyopathy [34–36] inherited cardiomyopathies [37, 38], valvular heart disease [39], cardiac transplant rejection [40]. In practice, at clinical field strengths (1.5 and 3 Tesla), a cardiac ^31^P spectrum is obtained from a 3D-localised myocardial voxel at specific acquisition times predefined by the user [41]. Acquisition times may vary with longer acquisition times required if small voxels and / or high signal to noise ratio is wanted [41]. ^31^P-MRS has inherently low signal to noise ratio and has low sensitivity to metabolites at low concentrations [41, 42]. This represents the main limitation of this technique as long acquisition time is required to achieve adequate signal to noise ratio [41, 42]. However, developments over the recent years are tackling this issue with the development of fast techniques mapping the phosphate metabolites [41].

**Fig. 4:**
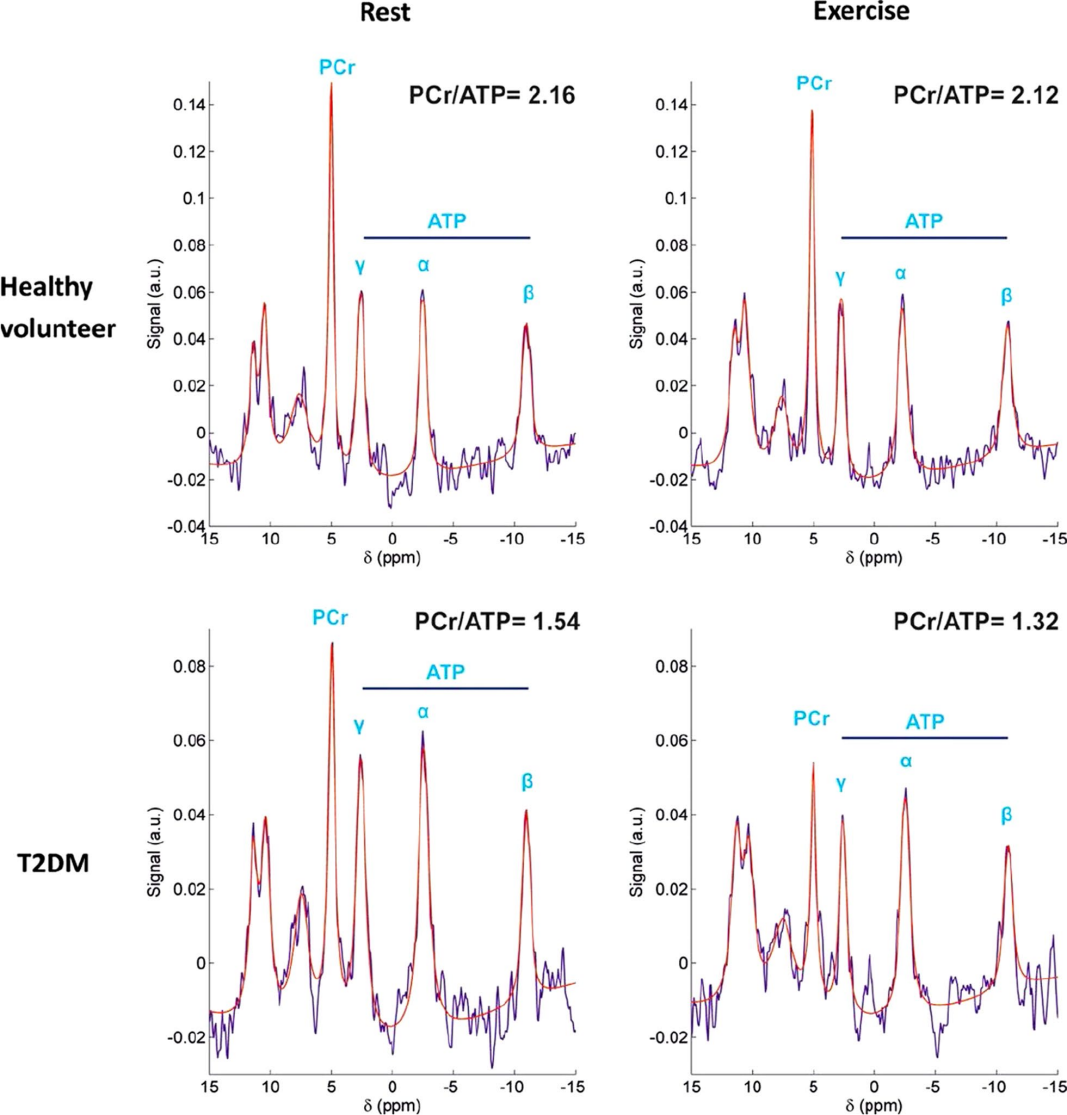
Rest and exercise myocardial 31P-MR spectra in a healthy volunteer (top row) and a T2D patient, suggesting a pre-existing energy deficit in the diabetic heart. Image reproduced with permission from Levelt et al. [47].

Over the last decade there has been notable progress in the field with the attempt to analyse the cardiac metabolic phenotype in detail non-invasively, and to delineate the relationship between the metabolic remodelling of the myocardium and the structural and functional changes. Due to the constantly varying cardiac workloads, efficient matching of energy supply to demand is essential for maintaining normal cardiac function [43] and myocardial metabolism is profoundly affected by changes in cardiac workload. The onset of exercise triggers a rapid increase in demand for substrate, and oxygen [44]. Rapid changes in the energy demand can be dealt by the fast responsive mechanisms of the healthy myocardium, [45], some of which include increased rates of phosphotransferase reactions [36, 46]. Assessing cardiac energetic response to exercise by ^31^P-MRS, aggravation of the pre-existing energetic insufficiency in patients with type 2 diabetes (T2D) was shown during increased workload (Fig. 4) [36]. Further, despite having no significant obstructive CAD, mean myocardial perfusion reserve index (MPRI) was significantly reduced in these patients [47, 48]. Pointing to the importance of an appropriate hyperaemic response during exercise to maintain cellular energy metabolism, significant correlations between MPRI with exercise energetics were demonstrated in patients with T2D [47]. While similar reductions in myocardial PCr/ATP during exercise was detected in patients with hypertrophic cardiomyopathy (HCM), in HCM this exacerbation of the energetic impairment was independent of perfusion reserve, as well as the degree of myocardial fibrosis or hypertrophy [37]. No significant changes in myocardial energetics with exercise activity was detected in comparison to the rest values in patients with dilated cardiomyopathy [49].

The CK system can also act as a buffer to diminish the differences in ATP and ADP levels, a feature which is crucial in maintaining the free energy of ATP hydrolysis in the cytosol. More recently, turnover rates of high-energy phosphates (CK Flux) in humans have also been measured with the saturation transfer method [50]. These dynamic turnover rates seem to be even stronger predictors of outcome in heart failure than steady-state concentrations [51]. Utilising this method, in a cohort of obese individuals and no other cardiac comorbidities, the myocardial CK reaction rate at rest was shown to be enhanced, preserving ATP delivery even with reduced PCr/ATP [52]. However, during augmented workload, ATP delivery through CK was not increased in the obese cohort in contrast with what is detected in non-obese controls suggesting that lower ATP delivery occurs during stress in obesity. This finding was shown to be associated with reduced systolic augmentation and exercise tolerance in patients with obesity. Highlighting the importance of myocardial energy supply through CK as a potential treatment focus to improve symptoms in obesity-related heart disease, weight loss has reversed these energetic changes. Similarly, the total CK flux capacity was recently shown to be reduced in patients with aortic stenosis (AS), with a reduced resting CK flux evident already in patients with moderate AS even with preserved LV systolic function [53]. These findings imply that moderate AS is associated with considerably impaired energetic state of the myocardium which is already established and a decline in the CK flux itself is not essential for the shift towards the systolic failure.

Finally, cellular metabolism can now by measured in vivo and in real-time thanks to the recent advancement of hyperpolarized 13C MRS.. Rider et al. successfully utilised hyperpolarized [1-13C] pyruvate MRS to assess downstream metabolism of [1-13C] pyruvate via PDH (pyruvate dehydrogenase, [13C] bicarbonate), lactate dehydrogenase ([1-13C] lactate), and alanine transaminase ([1-13C] alanine), in 5 patients with T2D and 5 controls at baseline, and repeated these measurements in 5 of these participants (3 T2D, 2 controls) 45 min after a 75 g oral glucose challenge [54]. They showed metabolic flux through cardiac pyruvate dehydrogenase (PDH) was noticeably declined in the individuals with T2D compared to controls. In addition, they have also detected a major increase in metabolic flux through PDH 45 min after the oral administration of 75 g of glucose in patients with T2D and in controls. This was the first study to show that physiological and pathological variations in PDH flux in the human heart can be evaluated with the use of hyperpolarized pyruvate, highlighting in this way that this method has the potential to assess metabolic shifts in a variety of cardiovascular disorders.

## Conclusion

The unique CMR methods discussed in this review are without doubt a pillar that can contribute substantially to future research and clinical practice.

USPIO CMR represents a robust novel method that assesses myocardial inflammation on a cellular level by identifying macrophage activity. It can be applied in a variety of diseases, including ischaemic and non-ischaemic cardiomyopathies, enabling in this way not only precise diagnosis and monitoring, but also targeted therapeutic interventions early in the disease process. MRS is another state-of-the-art tool ideal for the non-invasive assessment of cardiac energy metabolism, with its potential expanding in the evaluation of metabolic alterations in various different cardiovascular pathological processes. Further research and clinical studies are needed in order to explore how these novel CMR techniques compare with other imaging methods, such as positron emission tomography, and what clinical information they may add in the pathophysiological processes of a wide spectrum of cardiovascular diseases [55].

Considering that tissue inflammation and myocardial energetics play a key role in the pathophysiology of a wide range of cardiovascular diseases, these novel CMR methods and techniques are proving to meet the research and clinical expectations in assessing the disease processes on a cellular level. This, in turn, can provide clear insights not only in the diagnosis but also in the risk stratification and management strategies adapted in the future.

## Data Availability

Data sharing is not applicable to this article as no datasets were generated or analysed during the current study.

## Abbreviations

ADP: Adenosine diphosphate
AS: Aortic stenosis
ATP: Adenosine triphosphate
CABG: Coronary artery bypass graft
CAD: Coronary artery disease
CK: Creatine kinase
CMR: Cardiovascular magnetic resonance
HCM: Hypertrophic cardiomyopathy
LGE: Late gadolinium enhancement
LV: Left ventricular
MRS: Magnetic resonance spectroscopy
NSTEMI: Non ST segment elevation myocardial infarction
STIR: Short τ inversion recovery
STEMI: ST segment elevation myocardial infarction
T2D: Type 2 diabetes
^31^P-MRS: Phosphorus magnetic resonance spectroscopy
PDH: Pyruvate dehydrogenase
USPIO: Ultrasmall superparamagnetic particles of iron oxide
